# Screening for dementia with the Vienna Visuo-Constructional Test 3.0 screening (VVT 3.0 screening)

**DOI:** 10.1007/s40211-018-0279-9

**Published:** 2018-07-09

**Authors:** Noel Valencia, Johann Lehrner

**Affiliations:** Department of Neurology, Medical University of Vienna, Währinger Gürtel 18–20, 1097 Vienna, Austria

**Keywords:** Visuo-constructive functions, Subjective cognitive decline, Mild cognitive impairment, Alzheimer disease, Visuokonstruktive Funktionen, Subjektiver kognitiver Abbau, Leichte kognitive Beeinträchtigung, Alzheimer-Krankheit

## Abstract

**Background:**

Visuo-constructive functions are an important cognitive domain for the diagnosis and early detection of dementia. Using the Vienna Visuo-Constructional Test 3.0 Screening (VVT 3.0 Screening), we assessed visuo-constructive performance in subjective cognitive decline (SCD), mild cognitive impairment (MCI), Alzheimer’s disease (AD), and healthy control (HC) groups to determine whether VVT scores can be used to distinguish the mentioned diagnostic groups and predict disease progression to more advanced stages.

**Methods:**

We analyzed the data of 422 patients referred to the Department of Neurology, Medical University of Vienna, for assessment of neurocognitive status. We also examined 110 of these patients in a follow-up with regard to stability of performance and disease progression. We compared VVT performance across diagnostic groups and explored associations with relevant sociodemographic and clinical variables. Predictive validity was assessed using receiver operator characteristic (ROC) curves and multinomial logistic regression analyses.

**Results:**

We found that most diagnostic groups differed significantly regarding VVT scores. These were shown to reliably identify cases suffering from visuo-constructive impairment but were not sufficient for classification into all diagnostic groups. Progression to more advanced disease stages could not be reliably predicted using VVT scores, possibly because subsamples of progressors were quite small.

**Conclusion:**

VVT scores are useful indicators for identifying visuo-constructive impairment but are limited by factors such as similar disease manifestations when used to discriminate between several diagnostic groups. The same factors complicate the use of VVT scores for predicting disease progression to more advanced stages.

## Zusammenfassung

**Grundlagen:**

Eine der wichtigen kognitiven Domänen für die Diagnose und Früherkennung von Demenzerkrankungen sind visuokonstruktive Funktionen. In der vorliegenden Studie wurde die visuokonstruktive Leistung in den diagnostischen Gruppen subjektiver kognitiver Abbau (SCD), leichte kognitive Beeinträchtigung (MCI), Alzheimer-Krankheit (AD) und gesunde Kontrollen (HC) mithilfe des Vienna Visuo-Constructional Test 3.0 Screening (VVT 3.0 Screening) erfasst. Ziel war zu beurteilen, ob VVT-Punktwerte verwendet werden können, um diese diagnostischen Gruppen voneinander zu unterscheiden und den Fortschritt der Erkrankungen vorherzusagen.

**Methodik:**

Dazu wurden die Daten von 422 Patienten analysiert, die für eine Einschätzung ihres kognitiven Status an die Universitätsklinik für Neurologie der Medizinischen Universität Wien überwiesen wurden. Außerdem wurden 110 dieser Patienten in einer Verlaufskontrolle auf die Stabilität ihrer Leistungen und den Fortschritt ihrer Erkrankung untersucht. Die VVT-Leistungen wurden zwischen den diagnostischen Gruppen verglichen und Assoziationen mit relevanten soziodemografischen und neuropsychologischen Variablen geprüft. Die prädiktive Validität wurde mit Receiver-operating-characteristic(ROC)-Kurven und einer multinomialen logistischen Regressionsanalyse beurteilt.

**Ergebnisse:**

Die meisten diagnostischen Gruppen unterschieden sich signifikant hinsichtlich ihrer VVT-Punktwerte. Diese ermöglichten zwar eine zuverlässige Identifikation von Fällen, die an visuokonstruktiver Beeinträchtigung litten, erwiesen sich aber als unzureichend für die Klassifikation in alle vorhandenen diagnostischen Gruppen. Die Krankheitsprogredienz konnte anhand von VVT-Punktwerten nicht zuverlässig vorhergesagt werden, möglicherweise weil die Teilgruppen der Progressoren recht klein waren.

**Schlussfolgerung:**

Daraus schließen wir, dass VVT-Punktwerte zwar nützliche Indikatoren für die Identifikation visuokonstruktiver Beeinträchtigungen sind, die Unterscheidung zwischen mehreren diagnostischen Gruppen aber durch Faktoren wie ähnliche Krankheitserscheinungsformen erschwert wird. Die gleichen Faktoren verkomplizieren die Vorhersage der Progression zu weiter fortgeschrittenen Krankheitsstadien.

## Introduction

Dementia is a state associated with severe cognitive deficits and, as such, it substantially impairs intellectual, social and occupational functioning and features progressive and significant deterioration [1]. One progressive and irreversible disorder commonly leading to dementia is Alzheimer’s disease (AD), which is characterized by amyloid deposition, neurofibrillary tangles and the degeneration of cortical neurons and synapses. Some researchers have proposed that initial subjective cognitive decline (SCD) progresses gradually to mild cognitive impairment (MCI) and ends in dementia [2].

SCD describes a predementia phase in which patients remain within sociodemographically adjusted normal ranges while at the same time experiencing and expressing a subjective deterioration of cognitive abilities [2]. In contrast, during the transitional prodromal period of MCI, neuropsychological test performance reveals cognitive deficits which are greater than expected for the affected person’s age but do not yet cause the functional disruptions necessary for a dementia diagnosis [1, 3]. Since neurodegenerative changes are presumed to occur long before clinically noticeable problems become manifest [3, 4], reliable and valid instruments for early detection of SCD, MCI, and dementia are necessary as a basis for timely pharmacological intervention [5].

In this context, neuropsychological assessment has been shown to have some ability to predict the development of dementia before significant cognitive changes occur [2, 5]. One cognitive domain which several important clinical institutes include in their diagnostic guidelines for neurocognitive disorder (NCD) are visuo-constructive functions. The Diagnostic and Statistical Manual of Mental Disorders (DSM-5) [6], for instance, names perceptual-motor functioning as one of six cognitive domains which may be affected in NCD and includes visuoconstructional reasoning as one subcategory of this domain, and the National Institute on Aging—Alzheimer’s Association [7] lists visuo-constructive functions as an important criterion in its guidelines for the neuropathological assessment of MCI in Alzheimer’s disease. In addition to this, prior research has confirmed the value of measuring visuo-constructive functions when attempting to discriminate between subjects suffering from neurocognitive disorders and healthy controls [8, 9], and the fact that visuo-constructive decline can already occur in early stages of the disease [8] makes this domain important for screening instruments aimed at detecting incipient dementia.

The need for reliable instruments for assessing visuo-constructive functions led to the development the Vienna Visuo-Constructional Test (VVT; current version 3.0) [10] at the Medical University of Vienna. The test comprises three tasks which require copying three figures, namely a clock taken from the Montreal Cognitive Assessment (MoCA) [11], two overlapping pentagons taken from the Mini-Mental State Examination (MMSE) [12], and a three-dimensional cube taken from the Alzheimer’s Disease Assessment Scale (ADAS-Cog) [13]. (For a more thorough introduction to the VVT, see [9, 10].) Prior research [9, 14] demonstrated the value of the VVT in distinguishing between patients suffering from MCI, SCD, AD, and healthy controls (HC), respectively. The purpose of the present design was to build on these findings by replicating them with a larger sample and to further assess the prognostic validity of this instrument for early detection of dementia and for progression from SCD and MCI to AD. Specifically, we wished to assess the usefulness of the VVT 3.0 Screening version for these purposes, since it requires considerably less time than the full version. In this context, we expected those patients from the SCD and MCI groups who progress to AD to have lower initial scores on the VVT than those who do not progress. We also expected to find significant differences in VVT scores among all the mentioned groups except HC and SCD, as SCD patients usually do not yet perform worse than an age-adjusted norm group. In addition, we planned to reanalyze previously established associations between VVT and age, gender, premorbid intelligence quotient (IQ), global cognitive status, and depression [14]. Finally, the stability of VVT scores across examinations was also to be assessed in our design.

## Methods

The present analysis relies on data of patients living in Austria who participated in the Vienna Conversion to Dementia Study between 2008 and 2017. Written informed consent to use participants’ data in anonymized form for research purposes was obtained from all participants. Patients participated in an initial neuropsychological assessment and a subgroup also participated in one follow-up examination at a later date. The minimum time interval between examinations was set to 12 months and the maximum to 48 months so that some drop-out was expected.

## Instruments

Depression was measured using Beck Depression Inventory (BDI-II; [15]), and premorbid IQ was assessed using Wortschatz-Test (WST; [16]). The global cognitive status of patients was assessed with the MMSE [12]. The Neuropsychological Test Battery Vienna (NTBV) [17] was also administered in order to assess a broad range of cognitive domains, such as psychomotor speed, attention, language, memory, and executive functioning. We administered the VVT in the abbreviated screening version consisting of 10 items (three for the clock, three for the pentagons, and four for the cube), which Numrich [14] has shown to possess comparable criteria for test quality, namely Cronbach’s alpha 0.84 (full VVT 0.94), interrater reliability 0.84 (full VVT 0.90), sensitivity 0.94 (full VVT 0.84), and specificity 0.56 (full VVT 0.61). Administering the test usually requires between two and three minutes. Hereinafter, VVT 3.0 Screening scores will be referred to as “VVT scores” to improve readability.

## Sample characteristics

The sample we obtained for cross-sectional analysis consisted of 422 adult patients (183 men and 239 women) with a mean age of 68 years and a median MMSE score of 27. Descriptive statistics for the sample and diagnostic subgroups can be found in Table 1. Two patients with less than 8 years of schooling had previously been excluded from the analysis. The sample was made up of 53 patients classified as SCD, 218 patients as MCI, 122 patients as AD, and 29 in the HC group. Patients referred to the Department of Neurology at the Medical University of Vienna for assessment of cognitive functions are classified into diagnostic groups according to their performance on the NTBV during a neuropsychological evaluation coupled with a clinical interview. Specifically, SCD diagnoses were given following the guidelines of Jessen et al. [18], while Mayo clinic criteria [19] were applied for diagnoses of MCI and used to establish healthy functioning in HC [19, 20]. AD was diagnosed according to NICDS-ADRDA [21] and DSM-V criteria [6]. Visuo-constructive performance as measured by the VVT did not influence diagnosis.

**Table 1:** Relevant demographic and clinical characteristics in the sample and its subgroups

	Total	M/F	Age	MMSE
	*N*	*n*	Mean (range)	Median (range)
HC	29	13/16	52 (38–74)	29 (28–30)
SCD	53	28/25	65 (35–90)	29 (24–30)
MCI	218	95/123	68 (35–89)	28 (21–30)
AD	122	47/75	74 (41–94)	21 (7–27)
∑	422	183/239	68 (35–94)	27 (7–30)

*M/F* male/female, *MMSE* Mini Mental Status Examination, *HC* healthy controls, *SCD* subjective cognitive decline, *MCI* mild cognitive impairment, *AD* Alzheimer’s disease

**Fig. 1:**
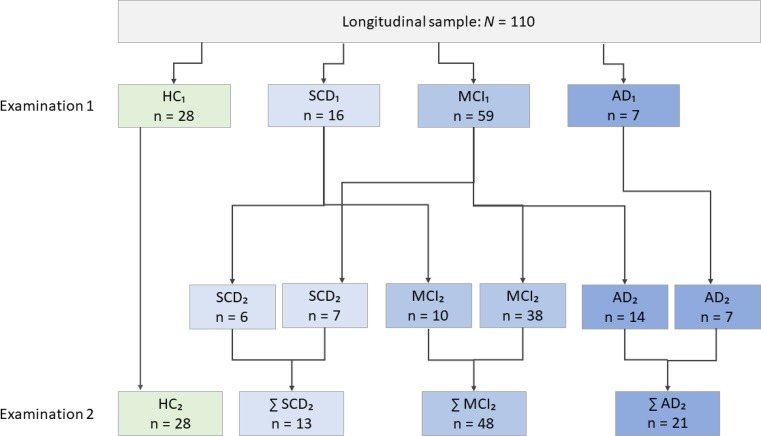
Patient flows between diagnostic groups across examinations. *HC* healthy controls, *SCD* subjective cognitive decline, *MCI* mild cognitive impairment, *AD* Alzheimer’s disease

Of the original 422 cases, 110 responded to our invitation and were assessed in a follow-up. The average time interval between examinations 1 and 2 was 21.93 months (standard deviation [SD] 10.16). This second sample consisted of 48 men and 62 women with an average age of 65.34 (SD 10.94). Distributions and patient flows across diagnostic groups are displayed in Fig. 1.

## Statistical analyses

Statistical analyses were conducted using IBM SPSS Statistics for Windows, Version 21.0 (IBM, Ehningen, Germany). Due to the considerable number of statistical tests in our design, we adjusted the alpha level of 0.05 using the Holm–Bonferroni correction^1^ in our analyses in order to control for the accumulation of type I errors. We chose this approach rather than the more conservative Bonferroni correction because of its higher statistical power and resulting lower type II error rate. As a result, significance for all *p *values reported hereinafter has been established in comparison with adjusted alpha levels (aa).

Since sample sizes in diagnostic groups differed considerably and VVT scores did not follow a normal distribution, nonparametric methods were applied for correlation analyses and group comparisons, whereas group medians and interquartile ranges (IR) are reported as descriptive statistics.

Predictive validity of VVT scores for distinguishing diagnostic groups was assessed using receiver operator characteristic (ROC) curves with AD as positive condition to determine sensitivity and specificity, and cut-offs were chosen according to the Youden Index. Following this, we calculated positive and negative predictive values (PPV/NPV), as well as positive and negative likelihood ratios (LR+/LR−). Further on, multinomial logistic regression was performed in the form of pairwise comparisons for a more detailed look into the discriminating ability of the VVT among the present diagnostic groups. We then calculated Cohen’s kappa coefficients to determine the degree of agreement between the classifications by the regression model and the diagnoses previously given by clinicians. We conducted Kruskal–Wallis analyses and pairwise Dunn’s post hoc comparisons to explore mean differences of VVT scores in all diagnostic groups. Due to postulated disease progression, one-tailed testing procedures were applied in all comparisons except HC-SCD.

Cross-tabulations were applied to assess progression rates. Here, too, ROC analyses were performed to evaluate the capability of VVT scores to predict these progressions. Differences between scores at examination 1 and 2 were explored using one-tailed Friedman tests. Following this, progressors and nonprogressors were compared using Mann–Whitney U tests. Due to large drop-out, in order to identify possible biases in our findings, we used Mann–Whitney U tests to compare those patients who participated in our follow-up at examination 2 with those who did not with regard to sociodemographic and clinical characteristics. We also conducted Spearman’s correlation analyses to explore associations between VVT scores and the mentioned sociodemographic and clinical variables.

## Results

Cross-sectional ROC analysis with AD as positive condition using VVT scores as predictor yielded an area under the curve (AUC) of 0.798 (95% confidence interval [CI] = 0.748–0.847, *p* < 0.001, aa 0.001). Based on the Youden Index, the most suitable cut off was determined at 8.50, resulting in a sensitivity of 0.60 and a specificity of 0.86. Based on this cut-off, PPV of 0.63 and NPV of 0.84 were computed together, with values of 4.18 and 0.47 for LR+ and LR−, respectively. An alternative cut-off maximizing sensitivity was identified at 9.5 and a sensitivity of 0.84 was attained together with a specificity of 0.59, PPV of 0.46 and NPV of 0.90, LR+ of 2.07, and LR− of 0.26. Finally, setting the cut-off at 7.50 in order to maximize specificity resulted in a sensitivity of 0.44 and a specificity of 0.94. In this case, PPV was determined at 0.75 and NPV at 0.81, as well as LR+ at 7.38 and LR− at 0.59.

The multinomial logistic regression model with VVT scores as predictor was specified significantly (*p* < 0.001, aa 0.001) with *Χ*^*2*^ = 122.99 and pseudo-*R*^*2*^ (Nagelkerke) = 0.28 for the entire sample with HC as reference category. The results of the pairwise group comparisons together with the odds ratios for classification into each diagnostic group in dependence of the set reference category are listed in Table 2. All pairwise group comparisons except HC-SCD and SCD-MCI yielded a significant *B*. Classification tables revealed that the regression model only made use of the two largest categories, predicting group membership in MCI for 82.90% of the cases and in AD in 17.10% of the cases. Thus, diagnostic group membership was predicted correctly for 92.70% of cases with an observed MCI diagnosis and for 44.30% of cases with an observed AD diagnosis, but none of the cases in HC or SCD were classified correctly. As a result, the regression model predicted diagnostic group membership correctly for 60.70% of cases in the total sample. The kappa coefficient was determined significantly (*p* < 0.001, aa 0.002) at 0.25.

**Table 2:** Parameters of multinomial logistic regression analysis with Vienna Visuo-Constructional Test (VVT) scores as predictors for diagnostic group membership

Reference category		*B* (SE) intercept	*B* (SE) VVT	Odds ratio
HC	SCD	5.05 (3.74)	−0.46 (0.38)	0.63 [0.30; 1.34]
	MCI	10.37 (3.63)*	−0.87 (0.37)*	0.42 [0.20; 0.87]
	AD	18.16 (4.14)*	−1.83 (0.43)*	0.16 [0.07; 0.37]
SCD	MCI	4.61 (1.77)*	−0.34 (0.19)	0.71 [0.50; 1.02]
	AD	10.77 (2.14)*	−1.13 (0.20)*	0.33 [0.21; 0.51]
MCI	AD	4.41 (0.73)*	−0.59 (0.08)*	0.55 [0.47; 0.65]

Model characteristics: *R*^*2*^ (Nagelkerke) = 0.28. Model *Χ*^*2*^ = 122.99, significant *p* < 0.001, aa 0.001

*Significance at the adjusted alpha level

*HC* healthy controls, *SCD* subjective cognitive decline, *MCI* mild cognitive impairment, *AD* Alzheimer’s disease, *SE* standard error, *aa* adjusted alpha

**Fig. 2:**
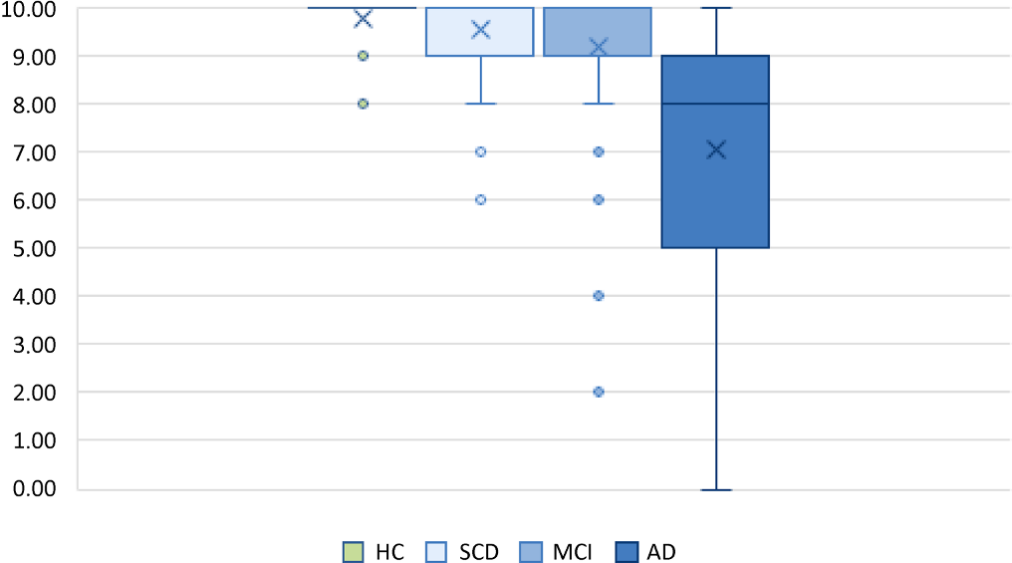
Vienna Visuo-Constructional Test (VVT) score distributions across diagnostic groups (× indicates the mean). *HC* healthy controls, *SCD* subjective cognitive decline, *MCI* mild cognitive impairment, *AD* Alzheimer’s disease

Kruskal–Wallis analyses using diagnosis as grouping variable revealed significant differences in VVT scores, with *χ*^*2*^(3) = 112.23, *p* < 0.001, aa 0.002, and mean ranks of HC 281.79, SCD 268.97, MCI 237.11, and AD 122.19. Subsequent one-tailed pairwise post hoc Dunn’s comparisons confirmed significant differences between HC and AD (*p* < 0.001, aa 0.002), SCD and AD (*p* < 0.001, aa 0.002), as well as MCI and AD (*p* < 0.001, aa 0.002). The remaining group comparisons HC-SCD (*p* = 0.217, aa 0.017), HC-MCI (*p* = 0.010, aa 0.005), and SCD-MCI (*p* = 0.035, aa 0.006) failed to reach significance. Score distributions in these diagnostic groups are also presented in Fig. 2.

A total of 16 cases with an SCD diagnosis at examination 1 had been assessed at examination 2 and were included in a longitudinal ROC analysis, setting MCI as the positive condition. AUC was determined at 0.525 ([95% CI = 0.229; 0.821], *p* < 0.001, aa 0.002). Using the Youden Index, the ideal cut-off was identified at 9.50 with a corresponding sensitivity of 0.20 and specificity of 0.83. Subsequently, PPV (0.67), NPV (0.38), LR+ (1.20), and LR− (0.96) were calculated. A sample of 59 cases which had received MCI diagnoses at examination 1 was the basis of a second ROC analysis with AD as positive condition. The resulting AUC was 0.537, (95% CI = 0.353; 0.722,* p* < 0.001, aa 0.002). The Youden Index suggested a cut-off of 8.50 with a sensitivity of 0.29 and a specificity of 0.82, and corresponding LR+ of 1.64 and LR− of 0.86. PPV was determined to be 0.33 and NPV to be 0.79.

One-tailed Friedman tests comparing VVT scores across examinations 1 and 2 failed to show significant differences in the total longitudinal sample: *χ*^*2*^ (1) = 4.08, *p* = 0.022, aa 0.005. Subsequently, we compared the VVT scores of progressors and nonprogressors with MCI diagnoses at examination 1 and repeated the same procedure for those who originally received SCD diagnoses. Neither the former comparison (*p* = 0.399, aa 0.013) nor the latter (*p* = 0.406, aa 0.017) yielded significant results.

Spearman’s rank correlations were used to explore the associations between VVT scores and relevant variables and revealed significant associations with gender (*r* = −0.17, *p* < 0.001, aa 0.003), age (*r* = −0.29, *p* < 0.001, aa 0.003), MMSE (*r* = 0.53, *p* < 0.001, aa 0.003), and WST-IQ (*r* = 0.24, *p* < 0.001, aa 0.003) but not with BDI-II (*r* = 0.53, *p* = 0.616, aa 0.050).

We also compared characteristics of one-time participants who did not respond to our invitation to a second assessment with those of follow-up participants who did, using Mann–Whitney U tests, and thereby determined that one-time participants were significantly older (*p* = 0.001, aa 0.004) and had significantly lower scores on VVT (*p* = 0.001, aa 0.004) and MMSE (*p* < 0.001, aa 0.003).

## Discussion

The present design evaluated the usefulness of neuropsychological assessment of visuo-constructive functions for (early) detection of dementia and prediction of disease progression. To answer our research questions, we assessed the predictive validity of VVT scores for distinguishing diagnostic groups at a first examination and to predict progression to a more advanced disease stage at a second examination.

Cross-sectional ROC analysis revealed an AUC of 0.798, and the optimal cut-off according to the Youden Index was determined at 8.50 with a corresponding sensitivity of 0.60 and specificity of 0.86. At this cut-off, 60% of patients were classified correctly as suffering from AD and 86% were classified correctly as not belonging to this diagnostic group. As shown by the AUC value, AD was identified well in the sample by VVT scores. If valid screening results are to be achieved, VVT screening must have a sufficiently high sensitivity, which is why an alternative cut-off value at 9.50 with higher sensitivity (0.84) could be considered even though it has a smaller corresponding Youden Index. The possibility of choosing alternative cut-offs for different questions make the VVT a very flexible instrument.

The LR values we determined only correspond to low levels of diagnostic validity [22], i. e., they may be important in some cases but not in the majority. Some confounding factors, such as disease severity and the presence of competing diseases with similar manifestations, have been shown to influence test accuracy [22]. To explore this possibility, the ability of VVT scores to discriminate between diagnostic groups was assessed using multinomial logistic regression and resulted in significant *B* coefficients in all pairwise comparisons except HC–SCD and SCD–MCI. However, the regression model only made use of MCI and AD in the classification procedures. In other words, when a case had a higher score on the VVT it was classified as MCI and when it had a lower score it was classified as AD. As a result, only 60.70% of cases in the total sample were classified correctly. When comparing the model classifications with the actual observed group membership, a low Cohen’s kappa coefficient of 0.25 was attained. We therefore conclude that there is not enough information in VVT scores to correctly classify cases into diagnostic groups, probably because scores are too similar in some groups. While MCI showed significant differences with regard to AD, it did not display these when compared to HC or SCD. It is thus likely that visuo-constructive decline in MCI is still too subtle to be picked up by a screening measure.

In general, VVT scores showed high heterogeneity in our cross-sectional sample and the respective diagnostic groups (Fig. 2). AD is a condition progressing over the course of several years and we included patients in very different stages of the disease in our sample. Some MCI and AD patients, for instance, displayed impairments in one, some in multiple cognitive domains measured by the NTBV. Similarly, visuo-constructive functions seem to have been almost intact in some cases and more impaired in others. As expected, HC and SCD did not differ significantly. Nonetheless, several outliers were present in these two groups as well. The reason why a few participants in the HC and the SCD group showed impaired visuo-constructive functions is not fully understandable. One might think of lack of motivation, lack of attention, or even low drawing abilities. However, this should be investigated further. Visuo-constructive performance in AD was significantly lower than in all other groups highlighting the ability of VVT to detect visuo-constructive impairment, which we expected to be highest in AD. Thus, the strongest feature of the VVT remains the ability to discriminate reliably between the presence and absence of AD.

Progression percentages were higher than reported in prior research [23, 24], with 62.50% from SCD to MCI (10 of 16 cases, see Fig. 1) and 23.70% from MCI to AD (14 of 59 cases). A considerable number of MCI patients who participated in our follow-up (7 patients) also regressed to the SCD group, highlighting the fact that, contrary to an AD diagnosis, this group is not considered to be subject to irreversible changes [1]. Furthermore, not a single case progressed from SCD directly to AD. This lends further support to the postulated development of dementia proceeding from SCD to MCI and eventually AD over the course of several years [2]. VVT scores in the total longitudinal sample were significantly lower at examination 2 mirroring the underlying disease progression. Though ROC analyses were conducted to ascertain the validity of VVT scores for predicting this disease progression, our results should be interpreted with caution since only 10 progressors from SCD to MCI and 14 from MCI to AD could be included in the analyses. In both analyses, AUC was determined at low levels (0.525 and 0.537, respectively) resulting in low sensitivity and specificity. This may have been due in part to the chosen time interval between examinations. On the one hand, given the large range of 12–48 months between examinations, some cases may have progressed more severely than others. On the other hand, in the majority of cases the time interval was not long enough to witness the entire course of the disease. Thus, the VVT’s predictive validity for progression may prove to be stronger in a design that covers a longer period. To sum up, our results should only be regarded as a first glance into the potential of predicting disease progression using visuo-constructive functions.

We also found moderate positive correlations between scores of VVT and MMSE. This is not surprising, as the MMSE provides an estimate of global cognitive functioning including visuo-construction. It can thus be concluded that, in a broader context, VVT scores are a useful indicator for the global cognitive status of patients, a conclusion which had already been drawn for other visuo-constructive instruments in previous studies [25]. Correlations with gender, age, and WST-IQ were negligible and no significant association with BDI-II scores was found.

## Strengths and limitations of this design

A few strengths and limitations of the present design should be taken into account when interpreting our findings. The sample we were able to recruit for cross-sectional analysis was quite large and featured the entire spectrum from healthy to severely ill. Another advantage was the comprehensive scrutiny that each case received for assessment of several cognitive domains. This approach resulted in detailed data and provided well-founded diagnoses. In contrast, our longitudinal sample was substantially smaller, which brings up the problem of drop-out bias (in fact, we found lower levels of global cognitive functioning in one-time participants). We assume that especially patients facing more severe disease progression failed to attend a second assessment, possibly due to related factors such as poor general health, which may have prohibited them from attending, or anosonognosia, which may have led them to believe a second assessment would not be necessary. In addition, our sample was selective to begin with as it only consisted of persons who actively approached us or were referred to us because of their complaints. Finally, an abbreviated version of the VVT 3.0 with somewhat different criteria for test quality was administered. Despite these drawbacks, we are confident that we have been able to contribute to a better understanding of visuo-constructive impairment and have added support to the value of VVT for the examination of it.

## Footnotes

1
$$\frac{\mathrm{target\,alpha\,level}}{\mathrm{number\, of\, tests}-\mathrm{rank\, number\, of\, pair}\left(\mathrm{by\, degree\, of\, significance}\right)+1}$$
